# Cancer Research Challenges and Potential Solutions in Saudi Arabia: A Qualitative Discussion Group Study

**DOI:** 10.1200/GO.23.00189

**Published:** 2024-01-02

**Authors:** Saleh A. Alessy, Abdulaziz A. Almotlak, Maha Alattas, Abdulraheem Alshareef, Kholoud Alwosaibai, Majed A. Alghamdi, Habeeb I.A. Razack, Saleh A. Alqahtani

**Affiliations:** ^1^ Public Health Department, College of Health Sciences, Saudi Electronic University, Riyadh, Saudi Arabia; ^2^ Division of Research & Innovation, King Faisal Specialist Hospital & Research Center, Riyadh, Saudi Arabia; ^3^ Centre for Cancer, Society & Public Health, Faculty of Life Sciences & Medicine, King's College London, London, United Kingdom; ^4^ Department of Pharmacology, College of Clinical Pharmacy, Imam Abdulrahman Bin Faisal University, Dammam, Saudi Arabia; ^5^ Department of Community Medicine, King Abdul-Aziz University, Jeddah, Saudi Arabia; ^6^ Department of Global Health, Boston University School of Public Health, Boston, MA; ^7^ Medical Laboratories Technology Department, College of Applied Medical Sciences, Taibah University, Madinah, Saudi Arabia; ^8^ Research Center, King Fahad Specialist Hospital, Dammam, Saudi Arabia; ^9^ College of Medicine, King Saud Bin Abdulaziz University for Health Sciences, Jeddah, Saudi Arabia; ^10^ Radiation Oncology, Princess Norah Oncology Center, King Abdulaziz Medical City, Ministry of National Guard Health Affairs, Jeddah, Saudi Arabia; ^11^ College of Medicine, King Saud University, Riyadh, Saudi Arabia; ^12^ Organ Transplant Center of Excellence, King Faisal Specialist Hospital & Research Center, Riyadh, Saudi Arabia; ^13^ Division of Gastroenterology & Hepatology, Johns Hopkins University, Baltimore, MD

## Abstract

**PURPOSE:**

Cancer incidence in Saudi Arabia has recently shown an upward trend. Research efforts within the different cancer continuum are pivotal to strengthening control measures. Since cancer research is evolving in the country, it is crucial to understand the current challenges and implement defined interventions to overcome them. The present qualitative study aimed to assess cancer research barriers among researchers and identify potential solutions from their perspectives.

**METHODS:**

We conducted a focus group discussion among 17 Saudi-based cancer researchers from diverse research backgrounds, provinces, and institutions. We used descriptive-interpretive thematic analysis following an open-ended approach to investigate the challenges in conducting cancer research. We also captured the solutions suggested based on the researchers' experiences.

**RESULTS:**

Six major themes emerged from the analysis: requirements of the data landscape, organizational support, national research roadmap, sustainable funding, clearer policies and regulations, and capacity building. To address challenges in these areas, researchers stressed the need for improved interinstitutional collaborations, immediate availability of research materials, and unlimited and easy access to research data.

**CONCLUSION:**

Improving health research is one of the primary goals of Saudi Vision 2030. It is, therefore, essential to overcome the current challenges in cancer research, enabling research findings to inform policies related to cancer control and care provision.

## INTRODUCTION

Cancer incidence has been increasing in Saudi Arabia (SA). The number of new cases is expected to increase from 27,885 in 2020 to 60,429 by 2040 (+116.7%).^1,2^ Research on the cancer continuum is pivotal in strengthening cancer control by generating country-specific evidence to guide effective contextual prevention, early detection, access to care, survivorship, and palliation.^3^ SA has invested massively in developing local research and higher education capacity through training initiatives and publicly funded scholarship programs.^4,5^ Besides, the Saudi Vision 2030 strategic framework emphasizes innovation and scientific research as top priorities for investment and development.^3^ Although cancer research in SA has progressed in the past three decades, research outputs from SA are still relatively lower than those from other Middle Eastern countries.^6,7^

**CONTEXT**

**Key Objective**
To understand the challenges and barriers that cancer researchers in Saudi Arabia (SA) encounter and document various solutions suggested by them.
**Knowledge Generated**
Cancer research challenges in SA have not been formally evaluated. The participants recognized the country's priorities that put cancer research at the forefront. However, they stressed the need for a clear, nationwide, dedicated strategy for cancer research alongside sustainable funding. In addition, the participants suggested the need for a timely increase in clinical trials and improved regulations favoring research in this domain.
**Relevance**
This study highlights the remaining challenges in cancer research in SA and identifies practical steps to unlock the current potential in the field. Thus, the findings and implications of this study are relevant to countries in the region, and many other countries that aim to improve cancer research.

Previous research in other contexts has identified challenges that researchers might face in cancer research settings, including the lack of funds, training, research regulations, cancer registration data, clinical studies infrastructure, or laboratory materials.^3,6,8^ To our knowledge, cancer research challenges in SA have not been formally evaluated. It is important to understand the challenges that may exist during the ongoing reform and the establishment of new national funding agencies, such as the Saudi National Institute of Health (SNIH)^9^ and the Research Development and Innovation Authority (RDIA),^10^ that aim to support research and innovation in SA.

The Saudi Society of Oncology Research (DAEM), a nonprofit organization, was established in 2021 to support cancer researchers.^11^ The present research was carried out as part of the DAEM's efforts to assess the current landscape of cancer research in SA. This qualitative study aimed to assess cancer research barriers researchers face and identify potential solutions from their perspectives. The evidence generated from this paper could contribute to strengthening the current cancer research capacity in SA.

## METHODS

This qualitative study used descriptive-interpretive thematic analysis following an open-ended approach to investigate how participants viewed the challenges they encountered conducting cancer research based on their experiences. Using purposive sampling, potential participants were invited from the DAEM email list (containing approximately 200 researchers). Additionally, an announcement was posted on Twitter, with several local researchers retweeting the post. Potential participants were asked to complete an online form (created using the Google Forms platform), and the information about their research activities and educational background was kept confidential. Participants were eligible if they (1) had 2+ years of experience and involvement in any area of cancer research in SA and (2) had at least one cancer-related research publication available in the scholarly domain. Two reviewers (S.A. Alessy and A.A.) examined the list to confirm eligibility for focus group participation.

Data were collected using a previously created focus group guide prepared by the study team. A 90-minute focus group discussion was conducted in English using the Zoom platform, and the recorded video was stored securely online (Google Drive). The group comprised all eligible participants, a moderator, and a note-taker. The principal investigators transcribed the entire recording. Two authors reviewed the transcripts and notes from the discussion, then coded the transcript text and extracted themes while considering repeated patterns. A codebook was created and regularly modified during the analysis to attain consensus on the meanings of the generated codes. Preliminary higher-level themes were identified in the following stage. Further refinement occurred after mapping the categorical themes. Data analysis was conducted using NVIVO (Version: 12 [released: March 2018]; QSR International, Burlington, MA).

The Institutional Review Board of Imam Abdulrahman Bin Faisal University reviewed and approved the study protocol (No. 2020-05-169). Voluntary participation and informed consent to use collected data for the research were ensured.

## RESULTS

Thirty-seven participants completed the online form, and 17 were deemed eligible for focus group discussion and ultimately participated. Table 1 provides participant demographic and background information.

**TABLE 1:**
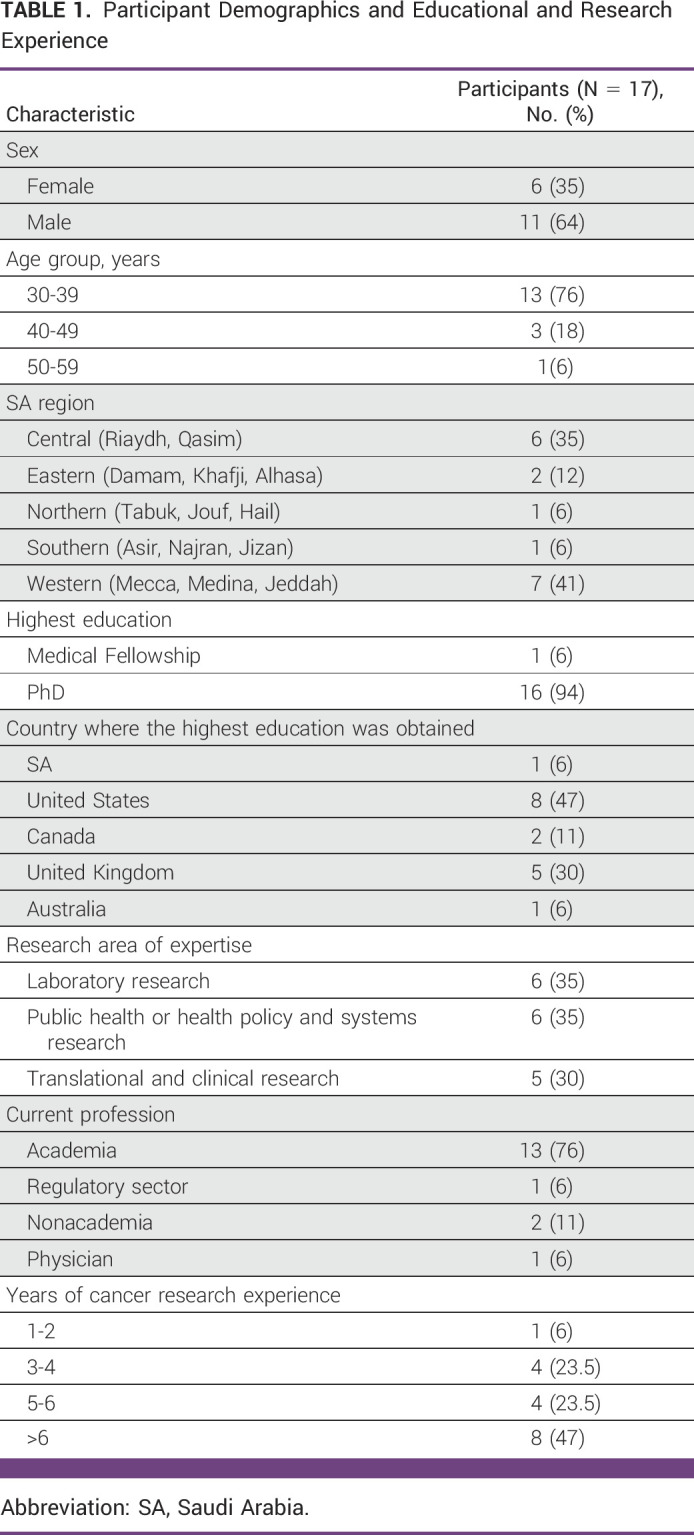
Participant Demographics and Educational and Research Experience

Eight themes were identified at the preliminary stage and five categorical themes were finalized. The invited researchers described challenges with various elements of the cancer research ecosystem, including data access, organizational structure, strategy and roadmap, policies, regulations, and funding availability. Several proposed strategies were raised during the discussion that, if properly implemented, could help decision makers improve the cancer research culture and environment in SA. Table 2 provides the selected verbatim conversations of participants on different aspects of this study. Table 3 summarizes the challenge categories, sub-barriers, and proposed solutions for each category.

**TABLE 2:**
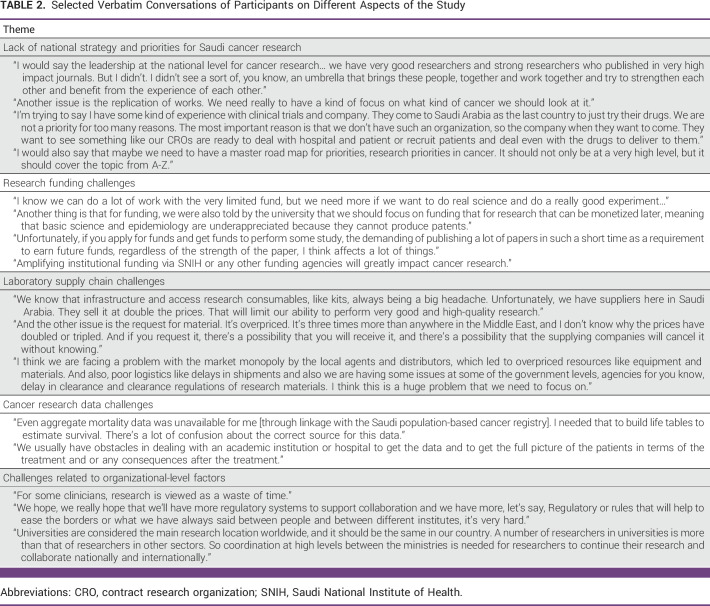
Selected Verbatim Conversations of Participants on Different Aspects of the Study

**TABLE 3:**
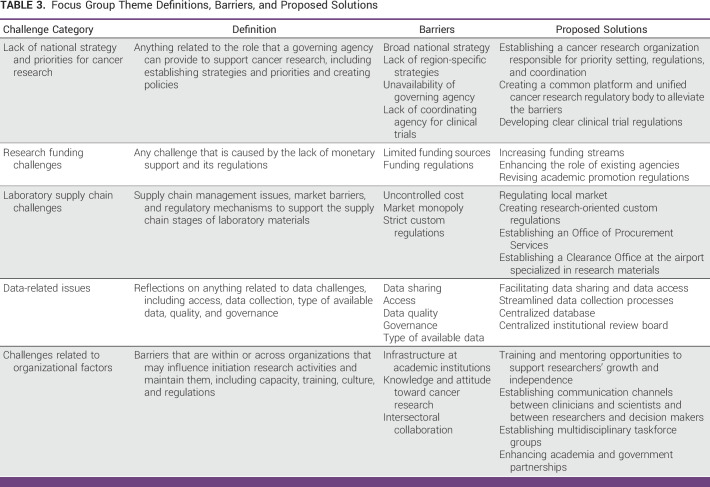
Focus Group Theme Definitions, Barriers, and Proposed Solutions

### Need for a National Strategy and Priorities for Cancer Research

Most participants recognized that the current national priorities put health and research at the forefront. However, many have noted a need for a clear nationwide cancer research strategy and regulatory agency for cancer research. One participant highlighted the importance of supporting regional research efforts (Table 2). Another mentioned the requirement of synthesized efforts that translate descriptive cancer data into actionable recommendations to guide cancer research ideas.

Developing a cancer research governing agency will strengthen the clinical trial environment. There are currently very few pharmaceutical company–sponsored clinical trials conducted in the Kingdom. Clear regulations and coordination between the company, the researchers, and the patients are needed.

#### Proposed Solutions

Participants recommended establishing a cancer research organization or strengthening the recently established Saudi National Cancer Institute to regulate and coordinate cancer-related research. Such an organization would also set a national roadmap for cancer research and develop national-level and provincial priorities to guide research activities.

Participants suggested a timely increase in clinical trials for better-tailored care for Saudi patients. Regulations should be improved to facilitate the environment for researchers and companies to conduct complex longitudinal research by developing clear clinical trial regulations and coordination pathways and incentivizing the global pharmaceutical industry. Participants believed this step would open the door for large-scale national and international collaborations.

### Research Funding Challenges

Current funding opportunities are limited. Government is the only source of financial support for cancer researchers in SA. Although private sector engagement in health care has gradually increased, its role in supporting cancer research is nonexistent. Most researchers expressed the dire need to increase research funding and improve the current retroactive payment process in which institutions ask researchers to pay for their laboratory materials out of pocket under the promise to be monitored based on research progress.

Multiple academic participants reported dissatisfaction with the funding regulations and guidelines (n = 5/17). Some institutions provide publication awards rather than funding from the beginning. This delayed funding mechanism rewards successful research but only encourages some research attempts. Participants stressed shifting the funding mindset toward research quantity over quality.

#### Proposed Solutions

Three participants suggested increasing funding streams by diversifying funding opportunities from the public and the private sectors. Others stressed the role that the SNIH, as a national agency, could play in funding cancer research. Researchers, especially in academia, suggested changing the academic promotional regulations linked to quantity over quality of research. Additionally, funding processes should be reviewed between the government and academia across relevant institutes. Funding allocation, size of awards, and publication requirements are among the changes that should be considered.

### Laboratory Supply Chain Challenges

Participants noted that purchasing laboratory materials is among the leading challenges for basic science research. Uncontrolled costs, market shortage, and distributors' unreliability regarding timely receipt of orders or price fluctuation affect the current supply chain management.

Researchers at wet laboratories shared their concerns about the local market's exorbitant laboratory equipment and material costs. Moreover, imported materials, such as biological specimens, often get delayed at customs because of the strict protocols, or lack thereof, to authorize shipment entry. These delays are affecting research progress and straining resources. For example, some materials that need controlling temperature lose their viability, burdening researchers, especially those paying for the materials out of pocket, given the restricted funding mechanisms.

#### Proposed Solutions

Improved standardized regulations for locally purchased materials may facilitate the work of basic science researchers. They also think agencies, such as the Saudi Food and Drug Authority may work with customs to facilitate a rapid entry pathway for sensitive research materials.

### Cancer Research Data Challenges

Issues on data revolved around data access, data governance, data type, and data quality. The participants repeatedly raised the need for more data as a major hindrance to conducting high-quality clinical research. The challenges cited varied and related to several themes, including data governance coordination, communication, and interinstitutional collaborations.

Participants mentioned challenges in using cancer-related research data because of accessibility issues. Agencies owning the data have to develop easy-to-use mechanisms for data requests. Application requirements imposed by institutions for data access should be relaxed and less bureaucratic. Explicit guidelines are needed to assist data requests. Additionally, for some domains, data ownership is scattered across multiple governing bodies, such as hospitals or health clusters, the Ministry of Health, or the Saudi Health Council, which creates administrative challenges. Finding ways for data linkages and harmonization with other national data sets must be accessible.

Moreover, the data collected at the institutional level may also be limited. Patient outcomes data must be routinely collected at every health care center with adequate quality checks. Multiple researchers faced challenges conducting prospective cohort studies and other studies requiring long-term follow-up. Data analysis and generating findings that could lead to scientific conclusions are also challenging. The limitation of the data collected could be attributed to scattered care, the absence of unified medical records, and variations in data quality.

#### Proposed Solutions

Researchers agreed that a more standardized and streamlined process should be developed to gain access to data in SA, especially genomic data and other national registries. Another suggestion was to strengthen the current Saudi Cancer Registry to link data from different sources with all data across cancer patients' care pathways, including laboratory tests, medications, progression of the disease, and mortality data. To tap into wider groups of populations, the private sector, including insurance companies, has to be involved by facilitating data sharing as an important stakeholder in advancing cancer research activities.

### Challenges Related to Organizational-Level Factors

Several barriers related to the research infrastructure, regulations, and capacity within academic institutions, government bodies, and health care facilities were identified. The research ecosystem at most academic institutions is not multidisciplinary within and across institutions. Participants stressed the need for sufficiently skilled workers to support high-quality basic science research or clinical trials. Some clinicians may not be interested in research. Besides, communication among researchers and other important stakeholders (eg, clinicians, administrative and executive leaders, etc) is challenging because of the bureaucracy created between organizations.

#### Proposed Solutions

To elevate the quality and efficiency of cancer research, additional training and mentoring opportunities are necessary to support researchers' growth and independence. Bolstering these types of programs would help to build local capacities to manage basic science cancer research laboratories. In addition, the role of academic institutions as partners to government agencies should be amplified.

## DISCUSSION

This study sought to assess the challenges of conducting cancer research in SA and the potential opportunities to overcome them. The study participants stated several challenges, including the lack of a national cancer research roadmap, research funding challenges, research logistics issues, and/or research barriers related to organizational factors.

Global cancer research has advanced greatly in the past two decades.^12^ Yet, variations in research capacities and challenges have been documented across countries,^8,13,14^ reflecting the need to support context-specific research for better global cancer control. Previous studies from the region have shown that cancer research outputs from SA are still relatively lower than those from other Middle Eastern countries.^6,7^ These challenges are more pronounced in translational research and clinical trials, a major area of research in developed countries.^6,7^ Besides, some challenges identified in our study have also been documented in some Middle Eastern and European countries.^15,16^ For example, the Lancet Oncology European Groundshot Commission on Cancer Research has recently identified many key recommendations to reimagine cancer research to deliver affordable, high-quality, better cancer care in Europe.^15^ Most were similar to those proposed in our study (eg, developing a national road map for cancer research, strengthening collaboration between institutions, improving the quality of cancer registration data, and increasing research funding). Moreover, some of the suggested solutions highlighted in this study have recently been implemented as part of the Saudi Vision 2030 goals, mainly through the recently established institutions (SNIH and RDIA). These initiatives include increasing and diversifying funding opportunities, building capacity, supporting clinical trials, and fostering research collaborations. Such initiatives have been shown to play a key role in promoting cancer research advancements and sustainability in many developed countries.^8,15,17,18^

Cancer is increasingly becoming a major health and economic burden in SA.^1,2^ Previous research studies have shown that countries must be research-active in achieving affordable and high-quality cancer care outcomes.^3,8,15^ Our study has several implications for policymakers, funding agencies, and research institutions. First, Saudi Vision 2030 highlights health research and innovations as one of the four pillars. Cancer research is vital in improving patients' lives. Evidence from the United Kingdom shows that cancer research can contribute to economic activities.^19^ A critical point raised by the participants in this study was to develop a national strategy. The recently established Saudi National Cancer Institute, hosted by the Saudi Health Council, is uniquely positioned to develop a roadmap for cancer research priorities in SA, which should align with the current National Cancer Control Plan to address the context-specific issues around the cancer burden in SA.^2,20,21^ Two successful cancer roadmaps include the Cancer Moonshot program in the United States, which announced in late 2016 and become the roadmap for the federal government; and the National Cancer Research Institute in the United Kingdom, which had been successful in attracting funding, prioritizing local issues, and advancing cancer sciences. The Saudi Health Council also hosts many national data sets, including the Saudi Cancer Registries. With the advancements in health data bioinformatics infrastructure in SA, it is important to propose initiatives to better cancer data collection from different sources, assure quality, and improve their accessibility to improve research on cancer occurrence patterns and care outcomes.^1,22,23^ Until recently, most of the health research in SA was produced at universities and funded through the Ministry of Education or King Abdulaziz City for Science and Technology,^24,25^ with some contributions from the private sector and nonprofit organizations. Recently, SNIH^9^ and RDIA^10^ have been established as a part of Saudi Vision 2030 to foster health research and innovation in the Kingdom through funding, coordinating, and regulating the research ecosystem, including cancer research.^26^

Another major obstacle was a complicated supply chain and procurement model for importing research materials. These issues mainly concern laboratory and translational research scientists and those whose research relies on advanced biotechnological products for which local manufacturers do not exist. To cope with this issue, localizing the biopharmaceutical industries and magnifying the local manufacturers' efforts to meet the country's demands are important. The new establishment of The National Industrial Development and Logistics Program should aim to enhance medical supply security, removing some challenges laboratory researchers face in SA.

Our paper has several important strengths. To our knowledge, this is the first-of-its-kind qualitative study that rigorously assessed cancer research capacity in the Kingdom using a focus group method. We included prominent researchers and discussed current challenges and strategies to improve cancer research. Participants were diverse in subject expertise, as many were trained in several well-established research countries, allowing for better exposure to identifying problems and providing solutions. A limitation of our study was the low participation rate of researchers in the study. Although we sent invites to approximately 200 researchers from the DAEM email list and posted invitation tweets, only 37 agreed to take part in the study, of whom 17 met the eligibility criteria. Further studies with larger sample sizes would help confirm the results obtained in the current study. Moreover, since the scope of this work was qualitative in nature, we did not intend to collect quantitative features, but future research is needed to collect more data from the cancer research workforce in SA including their backgrounds and inform local cancer research policies.

In conclusion, this study closely assessed the cancer research capacity in the Kingdom. Many significant challenges were identified. Organizational and policy changes in the research environment, capacity building and research skills training, and communication channels between professionals must be improved. As the country adopts Vision 2030, we are optimistic that several challenges will be overcome.
